# Volume Percentage of Filling Voids in Root Canals Prepared by a Novel Nickel-Titanium Rotary System (TruNatomy) Using Two Different Obturation Techniques

**DOI:** 10.3390/ma14143846

**Published:** 2021-07-09

**Authors:** You Jin Lee, Sunil Kim, Su-Jung Shin

**Affiliations:** 1 Gangnam Severance Hospital, Department of Conservative Dentistry, Yonsei University College of Dentistry, 211 Eonjuro, Gangnam-gu, Seoul 06273, Korea; yj1010@yuhs.ac; 2 Department of Conservative Dentistry and Oral Science Research Center, Yonsei University College of Dentistry, 50-1 Yonsei-Ro, Seodaemun-gu, Seoul 03722, Korea; seone1@yuhs.ac; 3 Gangnam Severance Hospital, Department of Conservative Dentistry and Oral Science Research Center, Yonsei University College of Dentistry, 211 Eonjuro, Gangnam-gu, Seoul 06273, Korea

**Keywords:** obturation, voids, TruNatomy, MicroCT

## Abstract

This study aimed to compare the volume percentage of filling voids in root canals prepared with a newly introduced rotary system, TruNatomy (Dentsply Maillefer), and obturated by the modified continuous wave (CW) or single cone (SC) filling technique. Plastic tooth models with four canals were enlarged by using TruNatomy files and randomly allocated into either the CW or SC group. The volume percentage of filling voids at 1–6 mm from the apex was analyzed by using microcomputed tomography; mean values were compared by using independent two-sample t-tests (*p* < 0.05). The mean volume percentages of the filling voids were 2.81 ± 1.11% and 1.77 ± 0.82% in the CW and SC groups, respectively. In the apical area (1–4 mm), volume percentages in the palatal were significantly different between the CW and SC groups; in the middle area (4–6 mm), volume percentages in the palatal and the second mesiobuccal canals were significantly different (*p* < 0.05). The SC group showed lower volume percentages of filling voids than the CW group. The canals prepared by the TruNatomy system can be obturated well by both the SC and CW techniques. The SC technique showed a lower number of voids, especially in the palatal canals.

## 1. Introduction

Utilizing the concept of minimally invasive endodontics, TruNatomy (Dentsply Maillefer, Ballaigues, Switzerland) has been recently introduced as a new generation of rotary nickel–titanium (NiTi) file system designed to preserve the maximum amount of peri-cervical dentine with a continuously tapering preparation [1,2]. The files are made of a new wire that has super-elastic properties; the manufacturer claims that this file system can follow the natural shape of the canal easily [2]. The apical tip sizes of TruNatomy files are similar to those of the other commonly used NiTi file systems. For example, the apical size of TruNatomy Prime Shaping file is 0.26, which is similar to that of ProTaper Gold F2 (Dentsply Maillefer) [3]. However, the TruNatomy files have a regressive taper and maintain a 0.8 mm maximum flute diameter. Although this new file system may have the advantage of less tooth preparation and preservation of tooth structure, it would be challenging for obturation in narrow canal spaces with less taper. For example, a less enlarged pericervical area will prohibit the heat plugger tip from reaching a location 3–5 mm from the working length if the continuous wave (CW) filling technique is used. To overcome this disadvantage, the manufacturer developed TruNatomy Conform Fit Gutta-Percha cones (Dentsply Maillefer). Based on the manufacturer’s instructions, these gutta-percha (GP) cones can be condensed at 7 mm from the working length by the heat plugger. By altering the thermoplasticity of the GP, they overcame the potential filling difficulty. Although the manufacturer suggested the compensatory filling method to overcome a limitation in filling narrow canals, there have been no studies to evaluate the filling quality in root canals using the TruNatomy system and this modified CW technique.

Moreover, it was questioned whether a single cone (SC) filling method that has become popular recently could be utilized for narrow canals prepared by TruNatomy files. The canals prepared by the TruNatomy NiTi file system are narrower than those prepared by other systems. When using the SC method with a calcium silicate–based sealer, there is a concern whether the calcium silicate–based sealer can penetrate the narrow canal sufficiently, because most calcium silicate–based sealers demonstrated lower flowability than epoxy-resin-based sealers such as AH Plus (Dentsply DeTrey, Konstanz, Germany) [4,5,6].

To the authors’ knowledge, there were previous studies that focused on the mechanical properties or canal shaping characteristics of the TruNatomy files [7,8,9]; however, none of these studies investigated the quality of obturation in root canals prepared by TruNatomy systems.

With this background, the purpose of this study was to measure the volume percentage of filling voids (%V) in canal fillings of artificial upper first molars prepared by using TruNatomy NiTi files and obturated by using TruNatomy Conform Fit Gutta-Percha cones by two different filling techniques and evaluated under micro-CT.

## 2. Materials and Methods

### 2.1. Preparation of Plastic Tooth Samples

Twenty-three plastic artificial teeth (Dentalike, Dentsply Maillefer, Ballaigues, Switzerland) with the shape of a human maxillary first molar were used. Each artificial tooth had four canals: first mesiobuccal (MB), second mesiobuccal (MB2), disto-buccal (DB), and palatal (P). MB and MB2 canals were classified as Weine classification type III [10]. To determine each canal’s working length (WL), a #10 sized K-file (Dentsply Maillefer) was placed in the canal, and the WL was determined until a point 0.5 mm from the apical tip.

All the canals were prepared by using the TruNatomy NiTi System (Dentsply Maillefer). TruNatomy Orifice Modifier, TruNatomy Glider, and TruNatomy Shaping Files were utilized with a speed of 500 rpm and a torque limit of 1.5 N/cm, according to the manufacturer’s instructions. The orifice modifier (#20, 0.08 taper) was used, and TruNatomy Glider (#17, 0.02 taper) was inserted into every canal up to the WL. For MB and DB canals, canal preparation was finished by using TruNatomy Prime Shaping file (#26). MB2 canal preparation was completed by using the TruNatomy Small Shaping file (#20), and P canal preparation was completed by using TruNatomy Medium Shaping File (#36).

**Figure 1:**
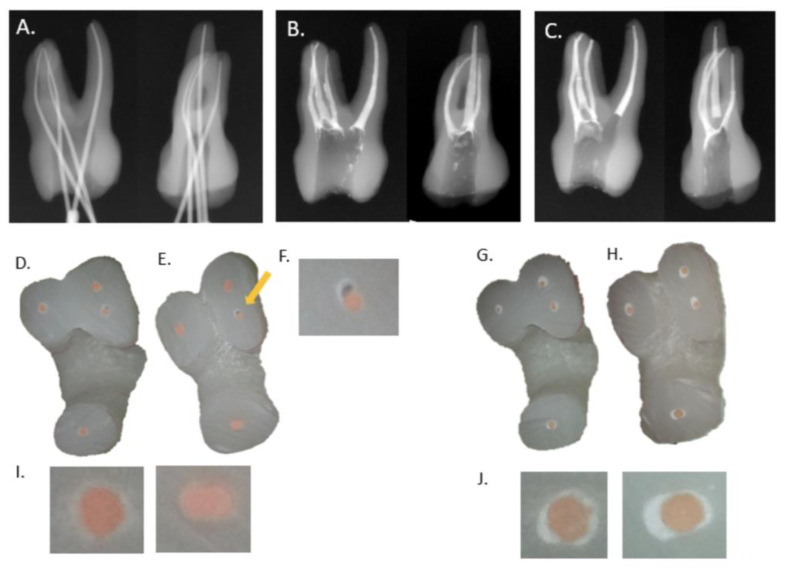
Radiographic and microscopic images of representative specimens. (**A**) Master cone fitting; MB (mesiobuccal canal), TruNatomy Prime; MB2 (the second mesiobuccal canal), Trunatomy Small; DB (distobuccal canal), TruNatomy Prime; P canal (palatal canal), TruNatomy Medium. (**B**,**C**) Periapical X-ray of a sample in SC (single cone filling) (**B**) and CW (continuous wave filling) (**C**) groups. (**D**,**E**) Microscopic image of canal filling at 3 mm (**D**) and 6 mm (**E**) from the apical tip of CW group. The yellow arrow indicates the void of the canal filling. (**F**) Magnified image of the void of the canal filling at 6 mm from the apical tip (**G**,**H**) Microscopic image of canal filling at 3 mm (**G**) and 6 mm (**H**) from the apical tip of SC group (**I**) Microscopic image of CW group of P canal (left, 3 mm; right, 6 mm). (**J**) Microscopic image of SC group of P canal (left, 3 mm; right, 6 mm).

After each filing procedure, the canals were irrigated by using distilled water and a TruNatomy irrigation needle (0.3 mm diameter) (Dentsply Maillefer). After completing canal instrumentation, the specimen was randomly assigned to one of the two groups based on the canal obturation technique. TruNatomy Conform Fit Gutta-Percha cones of matching size for each canal were placed into the canals and checked by radiography (Figure 1A). All canals were subsequently dried with fine-size paper points (DiaDent, Cheongju, Korea) before canal obturation.

### 2.2. Obturation of the Plastic Tooth Samples

The canal filling procedure was performed under a dental operating microscope.

- Modified continuous wave technique group (CW, *n* = 12): The GP cone tip was coated with a small amount of AH Plus (Dentsply DeTrey) sealer at the apical 3 to 4 mm part and inserted into the canal. The GP cone was cut at 7 mm from the WL, using a fine sized heat plugger (#30, 0.04 taper) (SybronEndo, Orange, CA, USA) and System B (SybronEndo), packed with BL S-Kondenser (#35) (B & L Biotech, Ansan, Korea) at the 7 mm level and backfilled by using SuperEndo Beta 2 (tip size #25) (B & L Biotech) at a temperature setting of 200 °C.
- Single-cone technique group (SC, *n* = 11): CeraSeal (Meta Biomed, Cheongju, Korea) was snugly inserted in the canal space by using the provided needle tip; the tip was gently moved towards the orifice from the point from which it was engaged in. A GP cone was moved upward and downward three times to ensure good penetration of the sealer, cut using a fine-sized heat plugger and System B (SybronEndo), and gently packed with BL S-Kondenser (B & L Biotech) at the orifice level.

All of the samples were stored in a special condition described in the previous study [11] for 14 days until further investigation, using micro-CT, to provide appropriate moisture for setting the calcium silicate–based sealer; this is because the sealer requires water for the setting reaction and adaptation to the root canal wall [5]. All procedures were performed by two experienced operators (S. J. Shin and Y. J. Lee).

### 2.3. Micro-CT Imaging and Analysis

Three artificial teeth samples were randomly selected by utilizing online random number generator program (Calculator Soup). The canal shape of three randomly selected artificial teeth samples before canal preparation was compared by scanning them with a micro-CT scanner (SkyScan 1173, Bruker, Billerica, MA, USA) to confirm the consistency of root canal space in the model teeth. The setting for imaging views by using the micro-CT scanner was the same as used in the previous study by Kim et al. [11].

After completing of canal instrumentation, all samples were scanned under the same conditions as mentioned above. Then, root canal filling was performed depending on the experimental groups; the samples were stored for two weeks and scanned again. The overlapped images were used for further analysis. Reconstructed images were obtained from the scan by using NRecon software version 1.7.0.4 (Bruker microCT, Kontich, Belgium). The range of measurements was 1–6 mm from the root apex, and the CT-An software (version 1.17.7.2, SkyScan) was used to measure the volume of the root canal space, that of GP, and that of the sealer. The area 1–4 mm from the apex constituted the apical area, and the area 4–6 mm from the apex constituted the middle area.

The %V was calculated as follows:

The method of this calculation was based on the previous studies by Kim et al. [11] and Jung et al. [12].

%V = {Vcanal − (Vsealer + VGP)}/Vcanal × 100(1)

where Vcanal is the volume of the canal and the Vsealer means the volume of the sealer, and the VGP means the volume of the gutta-percha. They were classified by grayscale.

### 2.4. Statistical Analysis

Sample calculation was first performed based on the results from a pilot study with a sample size of four in each group, using G*Power 3.1.9.6 (Universitat Kiel, Kiel, Germany) to detect significant differences (effect size: 1.27, alpha error: 0.05, power: 80%). The estimated sample size in each group was 11. To verify data normal distribution, Shapiro–Wilk and Kolmogorov–Smirnov tests were used. Independent two-sample t-tests were performed to compare the %V of the two different canal filling groups, using SAS version 9.4 (SAS Institute Inc., Cary, NC, USA). The *p*-values less than 0.05 were considered statistically significant.

After micro-CT scan, three representative samples from each group were selected for microscopic observation, and sectioned surfaces at the 3 and 6 mm levels were examined.

## 3. Results

The randomly selected three artificial tooth specimens were scanned to evaluate the consistency of canal shape and length among specimens; it indicated that the three artificial teeth had similar canal structures.

The microscopic images (20× magnification) of cross-sections at 3 and 6 mm from the apical tip revealed that both the SC and CW groups were well filled by the GP and sealer. The sealer portion of the SC group was greater than that of the CW group (Figure 1).

**Figure 2:**
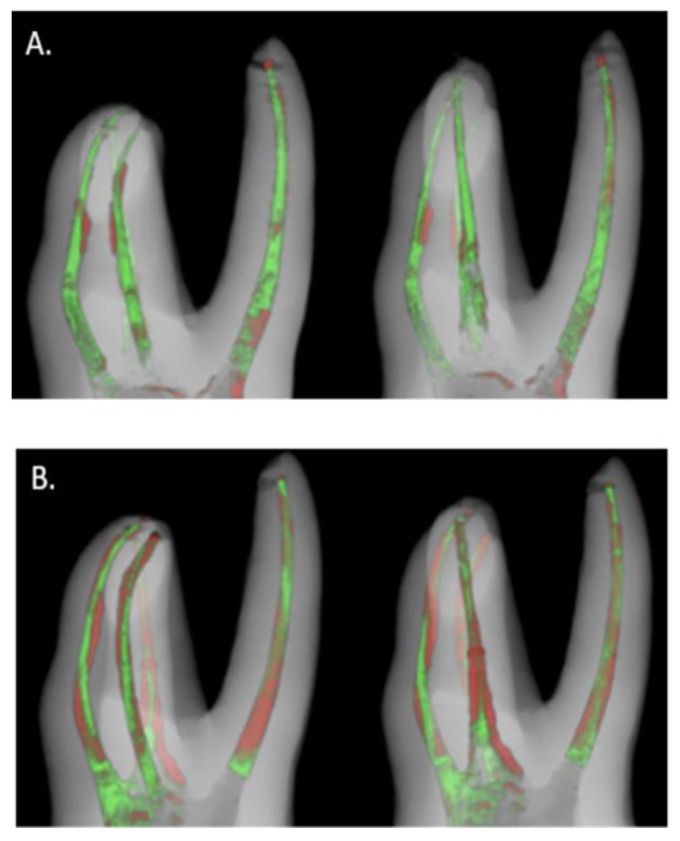
Three-dimensional reconstructed images of the obturated root canals. The green area indicates gutta percha and the red area displays sealer. The sample of the SC group showed more red space which means the sealer portion is more extensive than that of the CW group. On the other hand, gutta percha occupied most of the canal space of the CW group. (**A**) Reconstructed micro-CT scan image of CW group (**B**) Reconstructed micro-CT scan image of SC group CW, continuous wave filling; SC, single cone filling.

Canals of both the SC and CW groups were well filled with GP and the sealer as per micro-CT images. GP filled most of the canal space, and the sealer constituted the remaining space in the CW group compared with that in the SC group. In the area around 6 mm from the apical tip, the CW group showed more voids due to the gap between the surface of the cut master cone and the back-filled GP (Figure 2).

**Figure 3:**
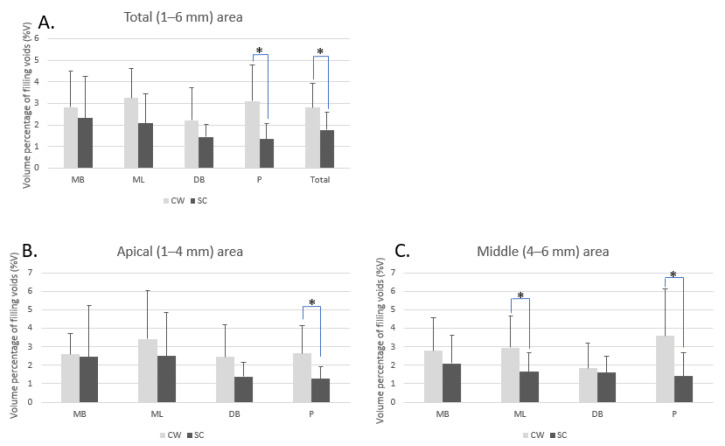
Bar graph of the volume percentage of canal filling voids. An independent two-sample t-test was performed to compare the void volume percent of the two different canal filling groups. (**A**) In the total area (1–6 mm from the apical tip), there is a statistically significant difference of the percentage of voids (%V) between CW (continuous wave filling group) and SC (single cone filling group) in P canal (palatal canals) and total canals. SC group showed less void volume percentage. (**B**) In the apical area (1–4 mm from the apical tip), the percentage of voids (%V) is significantly lower in the SC group in the P canal. (**C**) In the middle area (4–6 mm from the apical tip), the percentage of voids (%V) is significantly lower in the SC group in the MB2 (the second mesiobuccal canal) and P canals. Asterisks (*) indicate statistical significance (*p*-value < 0.05).

The mean values of %V in the CW and SC groups were 2. 81 ± 1.11% and 1.77 ± 0.82%, respectively, and there was a significant difference (*p* < 0.05). Based on the root canals, %V _total_ in the P canal demonstrated a significant difference between the two groups. In the apical area (1–4 mm), the %V_apical_ was significantly lower in the SC group in the P canal. In the middle area (4–6 mm), the %V_middle_ was significantly lower in the SC group in the MB2 and P canals (Figure 3).

## 4. Discussion

Our study compared the volume percentage of filling voids in canals prepared by three different sizes of TruNatomy files, using different filling techniques. The results demonstrated that both filling techniques used in the study showed favorable results.

In this study, the artificial teeth mimicking a maxillary first molar were used instead of human extracted teeth; many previous studies that used human extracted teeth were performed by using premolars or anterior teeth owing to the difficulty in controlling for the specimen since human molars have variations in the number of canals, anatomy, and length [13,14,15,16,17,18]. However, we considered that premolars and anterior teeth would have wider canal space than TruNatomy files. Hence, we used the plastic model teeth; however, the experiments using human teeth would be more relatable to clinical situations.

We compared two filling techniques that can be used to obturate narrow canals: CW and SC. The CW method used in this study was the modified method recommended by the manufacturer. Although it is more precise to use the same sealer in both groups, different sealers were used for each group in the experiment setting. Since the CW method contains heat application, a calcium silicate–based sealer was not used for this CW group due to a possible adverse effect of heat. Previous studies have reported that heat could affect calcium silicate–based sealers’ chemical and physical properties [19,20]. CeraSeal used in the SC group is one of the calcium silicate–based sealers designed for SC filling; however, the effects of heat application on the physical and chemical properties of the sealer have not been reported. Based on these reasons, AH plus (Dentsply DeTrey) was used in the CW group and CeraSeal (Meta Biomed) was utilized in the SC group.

Micro-CT and the volume of voids have been used to assess root canal filling quality [21,22,23]. It is a known fact that non-obturated areas may allow the persistence of bacteria at this site, resulting in treatment failure [13,24]. The analysis area was divided into the apical (1–4 mm) and middle (4–6 mm) portions. One millimeter from the apical tip was not included if the master cone did not go beyond apical 1 mm, because the void percentage could be overestimated. Thus, the study design was in agreement with other previous studies [11,25]. We also did not include the coronal area above 7 mm, since there might be a possibility of creating voids during backfilling, using the modified continuous filling technique.

Several previous studies analyzing the %V of root canal filling reported a range from 1.5% to 5.7%, and these values were consistent with our findings [11,13,14,16,26]. The %V was 3.61–5.72% in the SC and CW groups in artificial mandibular first molar teeth, as reported by Kim et al. [11]. By utilizing single-rooted teeth, void volumes of 1.57% and 1.61% were observed in the CW and SC groups, respectively, as demonstrated by Angerame et al. [26]. Our study demonstrated that, in the apical area, the SC group showed significantly lower %V in the P canal than in the CW group. In contrast, there was no significant difference with respect to the other canals. Based on these results, it is possible that the modified CW method used in this study might not result in proper adaptation of the thermoplasticized GP in P canals. However, the volume percentage of filling voids in the apical area in P canals were 2.64 ± 1.50% and 1.26 ± 0.69% in the CW and SC groups, respectively, and these values were comparable with those from the previous study by Kim et al. that used 3 mm downpacking [11]. In addition, the sectioned images and micro-CT scans demonstrated that the occupied portion of GP filling was greater in the CW group than in the SC group. These findings demonstrated that heat application of this modified method (GP cutting at 7 mm from the apical tip) efficiently delivers heat at the apical area in the CW group.

Another finding in our study was that, in the MB2 canal, it was challenging to apply the fine-sized heat plugger and condenser at the 7 mm level due to the width and curvature of the canal. The CW technique in the MB2 canal depends more on the operators’ proficiency as compared to the SC technique. Therefore, Chybowski et al. claimed that the SC filling method could be beneficial in filling narrow canals in this respect [27].

## 5. Conclusions

Our study demonstrated that root canals prepared by the TruNatomy file system could be well obturated by either modified CW or SC filling techniques. These results implicated that both techniques could be used in obturating the root canals minimally prepared. In the future, further research would be necessary to confirm the quality of obturation in minimally prepared canals in a clinical situation.

## Data Availability

The data presented in this study are available on request from the corresponding author.
